# Maesteg and District General Hospital, Maesteg

**Published:** 1919-10-11

**Authors:** T. M. Hollister

**Affiliations:** Matron.


*To the Editor of* THE HOSPITAL.

### Maesteg and District General Hospital, Maesteg.

*To the Editor of* THE HOSPITAL.

SIR,—There was an interesting note in your issue of last week regarding the district hospitals of Cardiff, and another note on the distribution of the Red Cross funds, in which mention was made of there having been no grant made to hospitals in one Welsh county. I believe the County Director for Glamorgan sent no application; consequently no hospital in this county has benefited by the distribution. There are several schemes under consideration in the various valleys round Cardiff for providing hospitals in the more populous districts, and so relieving the great pressure on King Edward VII. Hospital, where at present, I understand, there are over 2,000 waiting cases.

This hospital, from which I write, was built a few years ago to accommodate forty patients, in a mining town

**The Editor's Letter-Box**—(*continued*).

of 30,000 inhabitants, twenty-eight miles from Cardiff. Colliery accidents and all hospital cases had, until then, been sent to Cardiff or Swansea. The building was finished in 1915 and handed over entirely to the Red Cross Society for the reception of wounded soldiers; a small staff room being retained for the treatment of civilians as occasion demanded. During the occupancy by the Red Cross staff many defects in the structure and accommodation were discovered, and the place worked under disadvantages and difficulties.

It was demobilised in February last and taken over by the civilian committee. They have before them two alternative schemes. One is a plan for (1) adding small wards; this is very badly needed, as we have at present only two main wards of twenty beds each, one for men, the other for women and children. (2) Building a new theatre, the present one to be used as an out-patient dressing-room, as its ventilation, heating, and lighting are defective. (3) New kitchen and staff quarters, the present being quite inadequate. Also (4) a heating system. This plan at a cost of £12,000. The other scheme is a temporary one and strongly deprecated by the architect, costing £3,500. The population consists almost entirely of miners and tradesmen, and our difficulty is, of course, the question of funds. The conditions under which the Red Cross distribution was made are fulfilled by this hospital, inasmuch as we also treat ex-Service men. My disappointment on finding no grant had been made us was acute, as this hospital was the only one in Glamorgan which was entirely given over to the Red Cross. The larger building scheme is the only one the committee ought to consider; the lesser one is nothing but patching, and the difficulties under which the hospital is at present worked are great. We are usually full to overflowing—I frequently have to utilise a couple of beds in a recreation room. Since February our surgeons have performed fifty-one minor operations and eighty-four major ones, a total of 135. The institution is becoming very popular, the patients often being averse to leaving when convalescent, and when told their bed is required and they must go they are sometimes quite annoyed, and the situation has to be carefully explained to them. I feel this hospital is a real relief to the hard-worked institutions at Cardiff and Swansea, and in the near future we shall see more like it being built in the various mining valleys around Cardiff.—Yours truly,

T. M. Hollister (Matron).

*September* 30, 1919.

[We sympathise with Miss Hollister's disappointment over the non-allocation of Red Cross funds, which seems to be neither her fault nor that of the Red Cross. A point which arises out of her letter, but one upon which she does not touch, is how the various structural defects of her hospital came into being. Was the designing of this hospital thrown open to competition, or was it entrusted to a local architect of small experience in hospital planning? Welsh hospital committees are often too parochial in these matters; a form of local patriotism much less often seen in England, and one that certainly doesn't "pay."—Ed. The Hospital.]

